# DNA sequence polymorphisms in a panel of eight candidate bovine imprinted genes and their association with performance traits in Irish Holstein-Friesian cattle

**DOI:** 10.1186/1471-2156-11-93

**Published:** 2010-10-13

**Authors:** David A Magee, Klaudia M Sikora, Erik W Berkowicz, Donagh P Berry, Dawn J Howard, Michael P Mullen, Ross D Evans, Charles Spillane, David E MacHugh

**Affiliations:** 1 Animal Genomics Laboratory, UCD School of Agriculture, Food Science and Veterinary Medicine, University College Dublin, Belfield, Dublin 4, Ireland; 2 Genetics and Biotechnology Laboratory, Department of Biochemistry, University College Cork, Cork, Ireland; 3 Current address: Genetics and Biotechnology Laboratory, Centre for Chromosome Biology, National University of Ireland Galway, Ireland; 4 Animal and Bioscience Research Department, Animal and Grassland Research and Innovation Centre, Teagasc, Fermoy, Co. Cork, Ireland; 5 Animal and Bioscience Research Department, Animal and Grassland Research and Innovation Centre, Teagasc, Mellows Campus, Athenry, Co. Galway, Ireland; 6 Irish Cattle Breeding Federation, Highfield House, Bandon, Co. Cork, Ireland; 7 UCD Conway Institute of Biomolecular and Biomedical Research, University College Dublin, Belfield, Dublin 4, Ireland

## Abstract

**Background:**

Studies in mice and humans have shown that imprinted genes, whereby expression from one of the two parentally inherited alleles is attenuated or completely silenced, have a major effect on mammalian growth, metabolism and physiology. More recently, investigations in livestock species indicate that genes subject to this type of epigenetic regulation contribute to, or are associated with, several performance traits, most notably muscle mass and fat deposition. In the present study, a candidate gene approach was adopted to assess 17 validated single nucleotide polymorphisms (SNPs) and their association with a range of performance traits in 848 progeny-tested Irish Holstein-Friesian artificial insemination sires. These SNPs are located proximal to, or within, the bovine orthologs of eight genes (*CALCR*, *GRB10*, *PEG3*, *PHLDA2*, *RASGRF1*, *TSPAN32*, *ZIM2 *and *ZNF215*) that have been shown to be imprinted in cattle or in at least one other mammalian species (*i.e*. human/mouse/pig/sheep).

**Results:**

Heterozygosities for all SNPs analysed ranged from 0.09 to 0.46 and significant deviations from Hardy-Weinberg proportions (*P *≤ 0.01) were observed at four loci. Phenotypic associations (*P *≤ 0.05) were observed between nine SNPs proximal to, or within, six of the eight analysed genes and a number of performance traits evaluated, including milk protein percentage, somatic cell count, culled cow and progeny carcass weight, angularity, body conditioning score, progeny carcass conformation, body depth, rump angle, rump width, animal stature, calving difficulty, gestation length and calf perinatal mortality. Notably, SNPs within the imprinted paternally expressed gene 3 (*PEG3*) gene cluster were associated (*P *≤ 0.05) with calving, calf performance and fertility traits, while a single SNP in the zinc finger protein 215 gene (*ZNF215*) was associated with milk protein percentage (*P *≤ 0.05), progeny carcass weight (*P *≤ 0.05), culled cow carcass weight (*P *≤ 0.01), angularity (*P *≤ 0.01), body depth (*P *≤ 0.01), rump width (*P *≤ 0.01) and animal stature (*P *≤ 0.01).

**Conclusions:**

Of the eight candidate bovine imprinted genes assessed, DNA sequence polymorphisms in six of these genes (*CALCR*, *GRB10*, *PEG3*, *RASGRF1*, *ZIM2 *and *ZNF215*) displayed associations with several of the phenotypes included for analyses. The genotype-phenotype associations detected here are further supported by the biological function of these six genes, each of which plays important roles in mammalian growth, development and physiology. The associations between SNPs within the imprinted *PEG3 *gene cluster and traits related to calving, calf performance and gestation length suggest that this domain on chromosome 18 may play a role regulating pre-natal growth and development and fertility. SNPs within the bovine *ZNF215 *gene were associated with bovine growth and body conformation traits and studies in humans have revealed that the human *ZNF215 *ortholog belongs to the imprinted gene cluster associated with Beckwith-Wiedemann syndrome--a genetic disorder characterised by growth abnormalities. Similarly, the data presented here suggest that the *ZNF215 *gene may have an important role in regulating bovine growth. Collectively, our results support previous work showing that (candidate) imprinted genes/loci contribute to heritable variation in bovine performance traits and suggest that DNA sequence polymorphisms within these genes/loci represents an important reservoir of genomic markers for future genetic improvement of dairy and beef cattle populations.

## Background

Single nucleotide polymorphisms (SNPs) are the most abundant and widespread form of DNA sequence variation in vertebrate genomes [1]. By illustration, information on over 2.3 million pan-genomic SNPs has been generated via the analysis of the 7.1× bovine genome sequence assembly and this number is expected to increase with data from continuing re-sequencing projects [2,3]. Furthermore, as the vast majority of SNPs are biallelic they can be analysed using low-to-high throughput genotyping platforms, such as the BovineSNP50 assay [4], whereby SNPs are queried digitally for the presence or absence of a specific allele. These features have resulted in the rapid emergence of SNPs as the genetic marker of choice for the analysis of DNA sequence variation in single or small numbers of genes and whole livestock genomes [5].

High-density SNP data generated for livestock species using commercially available genotyping arrays have greatly enhanced the detection, mapping and characterisation of quantitative trait loci (QTL) for complex performance traits. However, in some cases the gene(s) or causative mutations underlying a particular QTL remain elusive because many SNPs included on genotyping platforms are located in non-coding regions of the genome. Therefore, animal geneticists often employ candidate gene strategies as viable alternatives to genome-wide scans for the detection of genes and DNA sequence/structural variation underling quantitative traits. The candidate gene approach uses variation in genes of known biological function relevant to the trait(s) of interest to investigate genotype-phenotype associations [6,7].

Previously, we adopted a candidate gene approach to detect genotype associations with performance in beef cattle by analysing SNPs in the bovine orthologs of genes shown to be imprinted in cattle or other mammalian species [8]. Genetic (or 'genomic') imprinting refers to the partial or complete transcriptional silence of one of the two parentally-inherited alleles that occurs in mammals in a parent-of-origin manner [9-11]. Genetic imprinting represents a recognisable form of epigenetic regulation in which chemical marks or "imprints", generally in the form of methyl groups (-CH_3_), are added to specific nucleotides across a gene sequence (*e.g*. CpG dinucleotides within the promoter sequence) during gametogenesis to regulate expression. These imprints are stably transmitted to the embryo and are further maintained in somatic cells with the pattern of imprinting for many of these genes being both developmental stage- and tissue-specific [12,13].

Studies in humans and mice have identified over 100 genes that are subject to imprinting and there is a substantial body of scientific evidence that highlights the importance of these genes in regulating mammalian development, metabolism and physiology [12-16]. More recently, there is accumulating evidence from studies in mammalian livestock species that polymorphisms within imprinted loci contribute to, or are associated with, heritable variation in several complex performance traits--most notably muscle mass, fat deposition, growth and milk production [17-27]. Additionally, there has been increased interest in the evolutionary consequences of imprinted loci in animal breeding systems and how parent-of-origin effects can be incorporated into statistical models for quantitative genetic analyses [28-31].

In the current study, we report our findings from analyses of genotype-phenotype associations between 17 validated SNPs distributed across eight candidate bovine imprinted genes/loci and genetic merit for a range of performance traits in progeny-tested Irish Holstein-Friesian dairy sires. These genes/loci are the calcitonin receptor gene (*CALCR*), the growth factor receptor-bound protein 10 gene (*GRB10*) [or maternally expressed gene 1 (*MEG1*)], paternally expressed gene 3 (*PEG3*), the pleckstrin homology-like domain, family A gene (*PHLDA2*), the RAS protein-specific guanine nucleotide-releasing factor 1 gene (*RASGRF1*), the tetraspanin 32 gene (*TSPAN32*), the zinc finger imprinted 2 gene (*ZIM2*), and the zinc finger protein 215 gene (*ZNF215*).

One of these genes (*PEG3*) has been previously shown to be imprinted in cattle [32,33], while the imprinting status of one bovine ortholog (*GRB10*) is equivocal [34]. No data regarding the imprinting status in cattle currently exist for five of these genes (*CALCR*, *PHLDA2*, *RASGRF1*, *TSPAN32 *and *ZNF215*); however, all five genes have been shown to be imprinted in one or more mammalian species (*i.e*. human/mouse/pig/sheep) [14-16]. Although species-specific imprinting has been documented previously for some genes [9,35], the appreciable conservation of genetic imprinting patterns between human and mouse [36,37] and humans and pigs [38] suggests that a proportion of the genes selected for analysis in the current study may also be imprinted in cattle. Furthermore, the documented molecular function of the encoded products of these genes suggests that they all play a pivotal role in mammalian growth and development and hence may represent potential and hitherto untested candidates for underlying variation in agro-economic traits.

The final gene analysed--zinc finger, imprinted 2 (*ZIM2*)--has recently been shown to be biallelically expressed in bovine testis tissue [32]. However, maternal imprinting of *ZIM2 *(*i.e*. expression from the padumnal allele) in humans and polymorphic imprinting of *ZIM2 *in mice (*i.e*. preferential maternal expression in brain tissue and biallelic expression in mouse testis), suggest a complex pattern of imprinting for this gene in different mammalian lineages [32]. *ZIM2 *forms an imprinted cluster or domain with the *PEG3 *gene in mammals [32,39] and this gene cluster has been implicated previously in playing a role in mammalian growth and development [40-42]. Consequently, SNPs within the bovine ortholog of the *ZIM2 *gene were included for the analyses presented here.

## Methods

### Single nucleotide polymorphisms (SNPs) selected for genotyping in the current study

A panel of 17 SNPs distributed across the bovine orthologs of eight genes (*CALCR*, *GRB10*, *PEG3*, *PHLDA2*, *RASGRF1*, *TSPAN32*, *ZIM2 *and *ZNF215*)--each of which have been shown to be imprinted in either cattle, human, mouse, pigs or sheep or more than one of these species--were selected for medium-throughput genotyping in this study. The ENSEMBL database (http://www.ensembl.org) accession for each of these genes together with their reported imprinted status in cattle or other mammalian species and the role of their encoded protein products are detailed in Table 1.

**Table 1:** The eight candidate bovine imprinted genes analysed in this study

Gene	Ensembl gene ID	BTA^1^	Gene function^2^	Imprinting status in cattle	Additional species in which gene is imprinted/Preferentially-expressed allele^3^
*CALCR*	ENSBTAG00000017458	4	Involved in regulating calcium homeostasis; involved in osteoclast-mediated bone resorption	No data available	Human; Mouse/ Maternal expression in both species

*GRB10*	ENSBTAG00000017086	4	Involved in signal transduction; interacts with insulin receptors and insulin-like growth factor receptors	Tested but unconfirmed^4^	Human; Mouse; Sheep/ Isoform-dependent maternal and paternal expression

*ZNF215*	ENSBTAG00000003638	15	Putative role in transcriptional regulation	No data available	Human/ Maternal expression but biallelic expression has been reported in some tissues

*PEG3*	ENSBTAG00000023338	18	Role in cellular apoptosis	Imprinted, paternal expression^5^	Human; Mouse; Sheep/ Paternal expression in all species

*ZIM2*	ENSBTAG00000011664	18	Putative role in transcriptional regulation	Biallelic expression^6^	Human; Mouse/ Paternal expression in humans. Tissue-specific maternal and biallelic expression has been reported in mouse

*RASGRF1*	ENSBTAG00000019940	21	Signal transduction and cellular proliferation	No data available	Mouse/ Paternal expression

*PHLDA2*	ENSBTAG00000031194	29	Tumour suppressor gene	No data available	Human; Mouse; Pig/ Maternal expression

*TSPAN32*	ENSBTAG00000002702	29	Possible tumour-suppressor functions	No data available	Mouse/ Developmental-stage specific maternal expression

Information regarding the expressed allele for all eight genes is based on the patterns of imprinting in mouse and human and, where possible, cattle, sheep and pigs. The chromosomal location of each gene was obtained from the ENSEMBL database (http://www.ensembl.org) and are based on Build 4.0, Ensembl release 59, of the *B. taurus *genome sequence (August 2010). The ENSEMBL database gene identity (ID) for each gene is given.

^1 ^*B. taurus *chromosome number

^2 ^Data taken from the GeneCards Version 3 database [64]; [http://www.genecards.org]

^3 ^Data taken from the Catalogue of Parent-of-Origin Effects database [16] and the Geneimprint database [14]

^4 ^Tveden-Nyborg *et al*. [34]

^5 ^Khatib *et al*. [35]

^6 ^Kim *et al*. [32]; Kim *et al*. [33]

Details for the 17 SNPs analysed in this study are presented in Table 2. Thirteen of these SNPs were previously validated via re-sequencing of high-fidelity polymerase chain reaction (PCR) products [8]. Information for three SNPs--distributed between the bovine *CALCR *gene (two SNPs) and *PHLDA2 *gene (one SNP)--was taken directly from the ENSEMBL database (these three SNPs were not validated by us via DNA re-sequencing previously or in the current study). The final SNP (*RASGRF1_p.C25039690T*) represents a *de novo *polymorphism located between the 7^th ^and 8^th ^exon of the bovine *RASGRF1 *gene on *Bos taurus *chromosome 21 (BTA21) and is presented for the first time here. This SNP was detected by sequencing a 1,095 base pair (bp) bovine *RASGRF1*-specific PCR product generated in a panel of 17 unrelated European *B. taurus *samples using amplification conditions detailed elsewhere [8] and two previously unpublished PCR primer sequences [forward primer: 5'-GCT TTC CTG AAT CTC TAT GC-3'; reverse primer 5'-TAG GAT TGA TGA GGT GAT CC-3'].

**Table 2:** Information for the SNPs genotyped in this study and summary statistics for the 848 genotyped Irish Holstein-Friesian AI sires

Gene/Expressed allele	BTA	SNP ID^1^	Nucleotide position of SNP	SNP gene position	Alleles (1/2)^2^	Frequency allele 1 (*p*)^3^	Heterozygosity	Deviation from HWE
*CALCR*/maternal	4	*rs42940189*	11,049,538	Exonic (non-syn)	G/A	0.90	0.15	< 0.001
	4	*rs42940187*	11,039,296	Exonic (syn)	C/T	0.86	0.23	0.03

*GRB10/* isoform-dependent	4	*GRB10_p.A5394141C*	5,394,141	Intronic	C/A	0.95	0.09	0.11
	4	*rs43375833*	5,334,910	Intronic	C/T	0.67	0.42	0.14

*ZNF215/*maternal	15	*rs42575466*	44,945,003	Exonic (syn)	G/A	0.95	0.09	< 0.00001
	15	*rs42575474*	44,934,196	Intronic	G/A	0.67	0.43	0.46

*PEG3/*paternal	18	*PEG3_p.A64370595G*	64,370,595	Upstream	G/A	0.69	0.44	0.36
	18	*PEG3_p.C64367437T*	64,367,437	Upstream	C/T	0.66	0.45	0.81
	18	*rs17871322*	64,362,259	Exonic (syn)	G/A	0.66	0.46	0.43

*ZIM2/* paternal expression in humans; polymorphic expression in mice	18	*rs41899915*	64,234,488	Exonic (syn)	C/G	0.80	0.32	0.43
	18	*rs41899913*	64,233,519	3'UTR	G/C	0.83	0.28	0.85
	18	*rs41899911*	64,232,216	3'UTR	C/T	0.80	0.32	0.41
	18	*rs41899910*	64,231,503	3'UTR	T/C	0.70	0.40	0.28

*RASGRF1/*paternal	21	*RASGRF1_p.C25039690T*	25,039,690	Intronic	A/G	0.59	0.42	< 0.001

*PHLDA2/*maternal	29	*rs42194502*	50,555,723	3'UTR	A/T	0.90	0.18	0.17

*TSPAN32/*maternal	29	*rs42637579*	51,123,847	Intronic	G/A	0.63	0.42	< 0.01
	29	*rs42637578*	51,123,729	Exonic (syn)	T/C	0.94	0.11	0.32

Genotype and allele frequencies and the significance of deviations from Hardy-Weinberg equilibrium (HWE) based on *P*-values obtained from χ^2^-test results are shown for all 17 SNPs. All SNP nucleotide positions were obtained from the Build 4.0 of the *B. taurus *genome sequence on the ENSEMBL database (http://www.ensembl.org) or the UCSC genome browser (http://genome.ucsc.edu). The ORF gene model positions for each SNP are given. For exonic SNPs, amino acid sequence changing SNPs (*i.e*. non-synonymous SNPs, denoted 'non-syn') and non-amino acid sequence changing SNPs (*i.e*. synonymous SNPs, denoted 'syn') are shown. The imprinting status of each gene is based on data from human and mice and, where possible, cattle, sheep and pigs [14,16].

^1 ^Where possible, SNP identities (IDs) are given as per their entry in the dbSNP database [43]; [http://www.ncbi.nlm.nih.gov/projects/SNP]. Where no dbSNP ID was available, SNPs were labelled as per nomenclature used by Magee *et al*. [8] as detailed in the main body text of the manuscript.

^2 ^Alleles 1 and 2 represent the major and minor alleles, respectively, at a given SNP

^3 ^The frequency of allele 1 (*p*), the major allele at a SNP locus

Where possible, SNPs were labelled in the present study based on their dbSNP database accession number [43]; [http://www.ncbi.nlm.nih.gov/projects/SNP]; however, four of the SNPs analysed here (*GRB10_p.A5394141C*, *PEG3_p.A64370595G*, *PEG3_p.C64367437T, RASGRF1_p.C25039690T*) were not deposited in the dbSNP database at the time of analysis. Instead, these four SNPs were re-coded according to the nomenclature adopted by Magee *et al*. [8]. For example, the *de novo RASGRF1_p.C25039690T *SNP was labelled whereby the gene associated with the SNP (*i.e*. *RASGRF1*) is reported first, followed by: (1) the symbol '*_p*.' which denotes a genomic DNA polymorphism; (2) the first allele at the SNP (*i.e*. a 'C' allele); (3) the nucleotide position of the SNP (*i.e*. 25,039,690) on BTA21 as per Build 4.0, release 59, of the *B. taurus *reference genome, and (4) the second allele at this locus (*i.e*. a 'T' allele). The *GRB10_p.A5394141C*, *PEG3_p.A64370595G *and *PEG3_p.C64367437T *SNPs were labelled in the same manner for this study.

Based on the current open reading frame (ORF) gene model reported for each gene in the ENSEMBL database, two SNPs were located upstream of the nearest gene, five SNPs were synonymous coding exonic substitutions, one SNP was a non-synonymous coding exonic substitution (resulting in an asparagine-to-aspartic amino acid substitution at amino acid position 116 of the *CALCR *gene), five SNPs were intronic and four SNPs were located in 3'UTRs (Table 2). All SNPs were biallelic and of these 13 were transitions (76.5%), while the remaining four were transversions (23.5%).

### Single nucleotide polymorphism (SNP) genotyping and the DNA samples analysed

All genotyping was performed by Sequenom Inc. (San Diego, CA, USA) using their proprietary MassARRAY iPLEX(tm) Gold platform (http://www.sequenom.com) and genomic DNA (gDNA) from 914 Irish Holstein-Friesian artificial insemination (AI) sires. gDNA from all 914 sires was extracted using a Maxwell(tm) 16 automated nucleic acid extraction apparatus (Promega Corp., Madison, WI, USA) according to manufacturer's instructions. The MassARRAY iPLEX(tm) Gold SNP genotyping platform discriminates between SNP alleles using single base primer extension technology after which primer extension products are analysed using matrix-assisted laser desorption ionization time-of-flight (MALDI-TOF) mass spectroscopy (http://www.sequenom.com/iplex). The 914 Holstein-Friesian sires have been used to generate progeny in Ireland and were representative of the commercial germplasm used in Irish dairy herds in recent years. For genotype quality control purposes, a panel of 25 independently-extracted, duplicate samples were also included for genotyping with the gDNA from 914 sires.

Genotype quality control and data filtering were performed on all data prior to association analyses. This involved the use of an iterative algorithm to remove SNPs and individuals that yielded poor genotype call rates. Firstly, SNPs with a genotype call rate 75% across all 914 individuals were removed, followed by the removal of individuals with genotype call rates of 85% across all remaining SNPs--this resulted in the removal of 21 sires and no SNPs from the study. Secondly, SNPs that yielded genotypes in 90% of all remaining 893 individuals were discarded followed by the removal individuals that failed to yield a genotype for 90% of all remaining SNPs--this resulted in the removal of a further 45 sires from the study, while no SNPs were discarded after the second filtering process.

After data filtering, genotypic data for all 17 SNPs and 848 progeny-tested sires with an average co-ancestry of 2.2% remained. A SNP genotype concordance rate of 99% between technical replicate samples was observed across all 17 SNPs; where discordance existed between the technical replicates the genotype for the sample in question was set to missing. Summary statistics for each SNP (including allele and genotype frequencies) and phenotype association analyses were performed using this edited dataset. *D*' [44] and *r*^2 ^[45] estimates of linkage disequilibrium (LD) between every pairwise combination of segregating SNPs within each gene/locus and imprinted gene cluster (*i.e*. the *PEG3 *imprinted gene cluster on *B. taurus *chromosome 15 [BTA15] that contains SNPs associated with the *PEG3 *and *ZIM2 *genes) were also generated from this edited dataset using the HAPLOVIEW software package [46].

### Phenotypic data and SNP genotype-phenotype association analyses

A range of phenotypic traits were analysed in this study and these were subdivided into seven broad categories: (1) *milk production traits *[milk yield, milk fat yield, milk protein yield, milk fat percentage and protein percentage]; (2) *udder/animal health *[somatic cell count]; (3) *carcass traits *[cow carcass weight, progeny carcass weight, progeny carcass (subcutaneous) fat level and progeny carcass conformation]; (4) *growth related traits in live animals *[animal stature, chest width, body depth, rump angle, rump width]; (5) *subjectively assessed subcutaneous fat level on live animals *[angularity and body condition score]; (6) *calving traits *[direct calving difficulty, maternal calving difficulty, perinatal mortality and calf survival]; and (7) *fertility *[gestation length and calving interval]. A detailed description of the phenotypic traits analysed in this study are provided in Additional File 1.

The phenotypes used in this study are sire genetic merit based not on data on the sires themselves but on the performance of their female progeny across multiple generations. Using known relationships among animals, performance records on relatives are used to estimate the genetic merit of an animal (*i.e. *a sire). Systematic environmental effects on the progeny are adjusted for and the random non-genetic variation associated with the progeny's phenotypes is minimised, thus facilitating a more accurate measure of genetic merit. This increased study power is particularly beneficial for low heritability traits where the proportion of phenotypic variance attributable to additive genetic differences is low. The disadvantage of such a study design is that the performance traits included for analysis are limited to those routinely measured on progeny. The average number of progeny per sire analysed here was 842 daughter-parity records. When coupled with the mixed model methodology used and the de-regression of the predicted transmitting ability (PTA), this implies that the associations reported herein are independent of pedigree structure.

Sire PTA was the dependent variable for all traits with the exception of the milk production traits, including somatic cell count, which were daughter yield deviations (DYDs) expressed on a PTA scale. Models used in genetic evaluations in Ireland, as well as variance components, have been previously described in detail [47] and summarised by Waters and colleagues [48]. All PTAs were de-regressed using the procedure outlined by Berry and colleagues [49]. Only sires with a reliability score, less parental contribution, of > 60% were retained for inclusion in the association analysis. A total of 742 sires fulfilled these criteria for inclusion in the analysis of milk, fat and protein yield as well as milk fat and protein concentration; the number of sires included in the association analysis with calving interval and survival was 501, and 477, respectively. The number of sires for direct calving difficulty, maternal calving difficulty, and perinatal mortality was 575, 506, and 201, respectively. The number of sires with a reliability of > 60% for the carcass traits was 446 and the number of sires with a reliability of > 60% for the size linear type traits varied from 484 to 551.

The association between each SNP and performance was quantified using weighted mixed linear models in ASREML [50] with individual included as a random effect, and average expected relationships among individuals accounted for through the numerator relationship matrix. Year of birth (divided into five-yearly intervals) and percent Holstein of the individual sire were included as fixed effects in the model. In all instances the dependent variable was de-regressed PTA or DYD, weighted by their respective reliability, less the parental contribution. Genotype was included in the analysis as a continuous variable coded as the number of copies of a given allele.

## Results

### SNP summary statistics

Summary statistics for each of the 17 SNPs assayed for this study are presented in Table 2. Minor allele frequencies (MAFs) for all SNPs were between 0.05-0.41. Heterozygosity (*i.e*. the proportion of heterozygous individuals) for all 17 SNPs ranged between 0.09-0.46, with a mean of 0.31 across all SNPs. Four SNPs displayed deviations from Hardy-Weinberg proportions (*P *≤ 0.01) and in each case this was due to an excess of homozygotes, presumably due to sampling error. Within-gene and within-gene cluster *r*^2 ^measures of LD (Additional File 2) ranged between 0.001 (for two pairwise SNP combinations within the *PEG3 *imprinted domain) and 1.000 (for the single pairwise SNP combination with the *ZIM2 *gene).

### A summary of SNP genotype-phenotype associations

Genotype association analysis identified nine SNPs that were associated (*P *≤ 0.05) with genetic merit for at least one of the performance traits assessed, while two SNPs--the single SNP in the *PHLDA2 *(*rs42194502*) gene and one in the *TSPAN32 *gene (*rs42637579*)--were not significantly associated with any of the traits analysed. The remaining six SNPs tended to be associated (*P *≤ 0.10) with at least one of the traits analysed. The genotype-phenotype associations detected in this study are discussed in further detail below.

### Associations with milk traits, somatic cell counts, calving traits and fertility traits

Allele substitution effects for milk traits, somatic cell counts and perinatal mortality are detailed in Table 3. None of the SNPs analysed were significantly associated with milk yield or milk fat yield (results not shown). A-to-G allele substitutions at the *rs42575474 *(*ZNF215 *gene) and *RASGRF1_p.C25039690T *SNPs were both associated (*P *≤ 0.05) with a reduction in milk protein percentage. The A-to-G allele substitution at the *RASGRF1_p.C25039690T *SNP was also associated (*P *≤ 0.05) with an increase in somatic cell score, while the A-to-G substitution at the *rs42575474 *SNP (*ZNF215 *gene) tended to be associated (*P *≤ 0.10) with an increase in somatic cell score. A tendency to be associated (*P *≤ 0.10) with milk traits was also observed at four other SNPs: the C-to-T allele substitution at the *rs42940187 *SNP (*CALCR *gene) with increased milk fat yield (+0.819 kg, standard error [SE] ± 0.466 kg) and no other SNPs were associated or tended to be associated with this trait, the *rs43375833 *SNP (*GRB10 *gene) with milk protein yield, the *rs42637578 *(*TSPAN32 *gene) SNP with milk fat percentage and milk protein percentage and the *rs42575466 *(*ZNF215 *gene) SNPs with milk protein percentage.

**Table 3:** SNP associations with milk traits, somatic cell score and calf perinatal mortality

SNP	Gene/BTA	Allele substitution	Milk protein yield (kg)	Milk fat percentage^1 ^(×100)	Milk protein percentage^1 ^(×100)	Somatic cell count^1 ^(units×100)	Calf perinatal mortality^1 ^(%×100)
*rs42940189*	*CALCR*/BTA4	A→G	0.073 (0.424)	-1.31 (1.15)	-0.47 (0.56)	-0.64 (0.92) 0.0.()	3.67 (14.79)
*rs42940187*	*CALCR*/BTA4	C→T	0.131 (0.365)	1.17 (0.99)	-0.07 (0.48)	0.78 (0.79)	-9.05 (12.35)
*GRB10_p.A5394141C*	*GRB10*/BTA4	A→C	0.103 (0.605)	-1.12 (1.64)	-1.03 (0.80)	0.43 (1.31)	-3.88 (21.17)
*rs43375833*	*GRB10*/BTA4	C→T	-0.522^†^ (0.280)	0.43 (0.76)	-0.08 (0.37)	0.04 (0.61)	7.99 (9.47)
*rs42575466*	*ZNF215*/BTA15	A→G	0.175 (0.575)	-0.73 (1.55)	-1.42^†^ (0.75)	0.28 (1.25)	-4.84 (19.26)
*rs42575474*	*ZNF215*/BTA15	A→G	0.009 (0.284)	-1.23 (0.77)	-0.77* (0.37)	1.13^†^ (0.61)	11.69 (9.28)
*PEG3_p.A64370595G*	*PEG3*/BTA18	A→G	0.127 (0.276)	0.14 (0.75)	-0.20 (0.37)	-0.77 (0.61)	0.09 (9.28)
*PEG3_p.C64367437T*	*PEG3*/BTA18	C→T	-0.025 (0.269)	-0.03 (0.73)	0.07 (0.35)	0.78 (0.58)	0.86 (9.35)
*rs17871322*	*PEG3*/BTA18	A→G	0.031 (0.278)	0.02 (0.75)	0.10 (0.37)	-0.72 (0.60)	-20.15* (9.61)
*rs41899915*	*ZIM2*/BTA18	C→G	0.031 (0.325)	0.71 (0.88)	-0.04 (0.43)	-1.01 (0.71)	20.64^†^ (11.32)
*rs41899913*	*ZIM2*/BTA18	C→G	0.303 (0.342)	-0.59 (0.92)	0.03 (0.45)	0.37 (0.75)	-25.22* (12.80)
*rs41899911*	*ZIM2*/BTA18	C→T	0.026 (0.326)	0.76 (0.88)	-0.03 (0.43)	-0.97 (0.71)	21.82* (10.97)
*rs41899910*	*ZIM2*/BTA18	C→T	-0.267 (0.293)	-0.03 (0.79)	-0.13 (0.39)	0.05 (0.64)	-2.33 (9.92)
*RASGRF1_p.C25039690T*	*RASGRF1*/BTA21	A→G	-0.104 (0.255)	-0.85 (0.69)	-0.70* (0.34)	1.29* (0.55)	1.55 (8.55)
*rs42194502*	*PHLDA2*/BTA29	A→T	0.087 (0.413)	0.64 (1.12)	-0.39 (0.54)	-0.40 (0.89)	24.19 (15.76)
*rs42637579*	*TSPAN32*/BTA29	A→G	0.078 (0.268)	-0.86 (0.72)	-0.33 (0.35)	0.35 (0.58)	-12.12 (9.24)
*rs42637578*	*TSPAN32*/BTA29	C→T	-0.776 (0.652)	-2.32^†^ (1.39)	-1.20^†^ (0.67)	-0.83 (1.11)	-29.91^†^ (15.55)

Standard errors for each trait are shown in parentheses. Levels of significance: ^†^*P *≤ 0.10; *P ≤ 0.05; **P ≤ 0.01. ^1^A value of 1 prior to multiplication by 100 equates to 1 percentage unit.

Three SNPs (*rs17871322 *[*PEG3 *gene], *rs41899913 *[*ZIM2 *gene], and *rs41899911 *[*ZIM2 *gene]) all within the *PEG3 *imprinted domain on BTA18 were associated (*P *≤ 0.05) with perinatal mortality, while the *rs41899915 *SNP (*ZIM2*) tended to be associated (*P *≤ 0.10) with this trait; none of the SNPs analysed were associated with calf survival. The low pairwise *r*^2 ^values of LD between the *rs41899913 *and *rs17871322 *SNPs (*r*^2 ^= 0.095) and the *rs41899911 *and *rs17871322 *SNPs (*r*^2 ^= 0.116) suggests that some of the observed associations with perinatal mortality and these *PEG3 *gene cluster SNPs are independent. In addition, the *rs17871322 *SNP within the *PEG3 *gene was the only SNP associated (*P *≤ 0.05) with both direct calving difficulty (*i.e*. maternal calving difficulty due to the size of the calf--a G-to-A allele substitution at this locus results in an increase in direct calving difficulty of 0.280%; SE ± 0.124) and maternal calf difficulty (*i.e*. a function of maternal pelvic width--a G-to-A allele substitution at this locus results in an decrease in calving difficulty of 0.289%; SE ± 0.144). The *rs17871322 *SNP was also the only analysed SNP to be associated (*P *≤ 0.05) with gestation length (an A-to-G allele substitution at this locus results in a decrease in gestation length of 0.154 days; SE ± 0.078). Collectively, these data point towards the *PEG3 *imprinted domain having a role in directing neonatal development. Finally, the T-to-C allele substitution at the *rs42940187 *SNP (*CALCR *gene) was negatively associated (*P *≤ 0.05) with calving interval (-0.664 days; SE ± 0.338) and was the only SNP to be significantly associated with this trait.

### Associations with carcass traits, fat deposition, body conformation and growth-related traits

The allele substitution effects associated with carcass traits, fat deposition on the live animal traits (angularity and body condition scores), body conformation traits and growth-related traits are detailed in Tables 4 and 5. Five SNPs (*rs42940187 *[*CALCR *gene], *GRB10_p.A5394141C*, *rs42575466 *[*ZNF215 *gene], *rs42575474 *[*ZNF215 *gene], and *rs41899913 *[*ZIM2 *gene]) were associated (*P *≤ 0.05) with angularity, while two SNPs (*rs42940187 *[*CALCR1 *gene] and *rs43375833 *[*GRB10 *gene]) were also associated (*P *≤ 0.05) with body condition score. Cow angularity and body condition score are genetically similar yet opposite traits and are subjective assessments of the subcutaneous fat deposits on a live animal [51]; lower angularity and greater body condition score indicates increased fat deposits.

**Table 4:** SNP associations with carcass traits and fat deposition traits

SNP	Gene/BTA	Allele substitution	Culled cow carcass weight (kg)	Progeny carcass weight (kg)	Progeny carcass conformation^1^	Progeny carcass fat^1^	Angularity^2^	Body condition score^2^
*rs42940189*	*CALCR*/BTA4	A→G	-0.880 (0.965)	-0.901 (0.747)	-0.030 (0.031)	-0.014 (0.027)	-0.154 (0.156)	0.104 (0.125)
*rs42940187*	*CALCR*/BTA4	C→T	0.738 (0.840)	0.877 (0.650)	0.012 (0.027)	-0.002 (0.024)	0.294* (0.129)	-0.239* (0.103)
*GRB10_p.A5394141C*	*GRB10*/BTA4	A→C	-2.044 (1.529)	-0.453 (1.191)	0.004 (0.049)	0.071^†^ (0.043)	0.435* (0.216)	-0.052 (0.171)
*rs43375833*	*GRB10*/BTA4	C→T	-0.333 (0.641)	0.623 (0.498)	0.047* (0.021)	0.002 (0.018)	-0.113 (0.097)	0.171* (0.078)
*rs42575466*	*ZNF215*/BTA15	A→G	2.118 (1.294)	1.394 (0.996)	-0.071^†^ (0.042)	-0.020 (0.037)	0.506* (0.217)	-0.180 (2.584)
*rs42575474*	*ZNF215*/BTA15	A→G	1.882** (0.648)	1.168* (0.503)	0.0174 (0.021)	-0.030^†^ (0.018)	0.290** (0.095)	1.805 (1.183)
*PEG3_p.A64370595G*	*PEG3*/BTA18	A→G	-0.065 (0.644)	-0.257 (0.500)	0.006 (0.021)	-0.030^†^ (0.018)	0.009 (0.091)	-0.045 (0.073)
*PEG3_p.C64367437T*	*PEG3*/BTA18	C→T	0.180 (0.643)	0.095 (0.499)	-0.024 (0.021)	0.023 (0.018)	0.028 (0.090)	0.018 (1.145)
*rs17871322*	*PEG3*/BTA18	A→G	-0.624 (0.648)	-0.490 (0.505)	0.032 (0.021)	-0.005 (0.018)	-0.075 (0.091)	0.044 (1.138)
*rs41899915*	*ZIM2*/BTA18	C→G	0.104 (0.767)	-0.331 (0.596)	-0.020 (0.025)	-0.011 (0.022)	-0.081 (0.106)	0.987 (1.295)
*rs41899913*	*ZIM2*/BTA18	C→G	0.131 (0.832)	0.332 (0.651)	-0.013 (0.027)	0.006 (0.024)	0.244* (0.112)	0.636 (1.409)
*rs41899911*	*ZIM2*/BTA18	C→T	0.120 (0.766)	-0.321 (0.596)	-0.024 (0.025)	-0.010 (0.022)	-0.059 (0.106)	0.946 (1.291)
*rs41899910*	*ZIM2*/BTA18	C→T	0.527 (0.685)	0.101 (0.531)	0.015 (0.022)	-0.005 (0.019)	0.121 (0.096)	-0.891 (1.158)
*RASGRF1_p.C25039690T*	*RASGRF1*/BTA21	A→G	-0.375 (0.584)	0.414 (0.459)	0.035† (0.019)	0.003 (0.017)	-0.154^†^ (0.085)	-1.540 (1.056)
*rs42194502*	*PHLDA2*/BTA29	A→T	0.064 (0.975)	-0.322 (0.762)	-0.018 (0.032)	0.007 (0.027)	0.026 (0.135)	0.178 (1.678)
*rs42637579*	*TSPAN32*/BTA29	A→G	-0.076 (0.642)	-0.504 (0.504)	-0.023 (0.021)	0.015 (0.018)	0.100 (0.090)	0.499 (1.130)
*rs42637578*	*TSPAN32*/BTA29	C→T	1.918^†^ (1.147)	0.757 (0.899)	0.031 (0.038)	-0.054 (0.039)	-0.022 (0.172)	-1.055 (2.076)

Standard errors for each trait are shown in parentheses. Levels of significance: ^†^*P *≤ 0.10; *P ≤ 0.05; **P ≤ 0.01. Progeny carcass fat score and progeny carcass conformation score are shown on a scale of 1.00 (low) to 15.00 (high) according to Hickey *et al*. [97]. ^1 ^A value of 1 equates to 1 percentage unit. ^2 ^Expressed in genetic standard deviations.

**Table 5:** SNP associations with body conformation traits and growth-related traits

SNP	Gene/BTA	Allele substitution	Body depth^1^	Rump angle^1^	Rump width^1^	Stature^1^
*rs42940189*	*CALCR*/BTA4	A→G	-0.240^†^ (0.140)	0.136 (0.149)	0.062 (0.157)	-0.169 (0.151)
*rs42940187*	*CALCR*/BTA4	C→T	0.161 (0.116)	-0.050 (0.125)	-0.151 (0.130)	0.169 (0.124)
*GRB10_p.A5394141C*	*GRB10*/BTA4	A→C	0.166 (0.193)	0.191 (0.198)	0.352^†^ (0.206)	0.371^†^ (0.200)
*rs43375833*	*GRB10*/BTA4	C→T	-0.001 (0.088)	-0.239* (0.093)	0.067 (0.097)	-0.033 (0.094)
*rs42575466*	*ZNF215*/BTA15	A→G	0.280 (0.195)	0.075 (0.200)	0.173 (0.206)	0.479* (0.202)
*rs42575474*	*ZNF215*/BTA15	A→G	0.245** (0.086)	-0.018 (0.091)	0.293** (0.093)	0.302** (0.092)
*PEG3_p.A64370595G*	*PEG3*/BTA18	A→G	-0.106 (0.083)	0.109 (0.087)	-0.156^†^ (0.092)	-0.082 (0.088)
*PEG3_p.C64367437T*	*PEG3*/BTA18	C→T	0.152^†^ (0.081)	-0.053 (0.086)	0.140 (0.090)	0.111 (0.087)
*rs17871322*	*PEG3*/BTA18	A→G	-0.180* (0.083)	0.068 (0.088)	-0.168^†^ (0.092)	-0.212* (0.088)
*rs41899915*	*ZIM2*/BTA18	C→G	-0.101 (0.096)	0.119 (0.101)	-0.046 (0.105)	-0.113 (0.102)
*rs41899913*	*ZIM2*/BTA18	C→G	0.183^†^ (0.101)	-0.114 (0.107)	0.154 (0.112)	0.250* (0.108)
*rs41899911*	*ZIM2*/BTA18	C→T	-0.084 (0.096)	0.107 (0.101)	-0.031 (0.106)	-0.088 (0.103)
*rs41899910*	*ZIM2*/BTA18	C→T	0.123 (0.087)	-0.148 (0.091)	0.048 (0.095)	0.120 (0.093)
*RASGRF1_p.C25039690T*	*RASGRF1*/BTA21	A→G	-0.066 (0.076)	0.012 (0.0801	-0.075 (0.084)	-0.096 (0.081)
*rs42194502*	*PHLDA2*/BTA29	A→T	-0.131 (0.122)	0.210 (0.128)	-0.081 (0.135)	0.001 (0.130)
*rs42637579*	*TSPAN32*/BTA29	A→G	0.027 (0.082)	-0.008 (0.085)	0.044 (0.089)	0.075 (0.087)
*rs42637578*	*TSPAN32*/BTA29	C→T	0.062 (0.156)	0.124 (0.161)	-0.150 (0.170)	0.079 (0.165)

Standard errors for each trait are shown in parentheses. Levels of significance: ^†^*P *≤ 0.10; *P ≤ 0.05; **P ≤ 0.01. ^1 ^Expressed in genetic standard deviations.

Five SNPs were associated (*P *≤ 0.05) with at least one of the carcass traits or animal growth traits assessed. An A-to-G allele substitution at the *rs42575474 *(*ZNF215 *gene) SNP was associated with gains in progeny carcass weight (*P *≤ 0.05) and culled cow carcass weight (*P *≤ 0.01); no other SNPs were associated with these traits (results not shown). Similarly, the A-to-G substitution at this locus was also associated with greater body depth (*P *≤ 0.01) and taller animals, as illustrated with associations with animal stature (*P *≤ 0.01) with wider rumps (*P *≤ 0.01), suggesting that this gene plays a role in promoting growth. The other genotyped *ZNF215 *SNP (*rs42575466*) also displayed an association (*P *≤ 0.05) with animal stature. Both *ZNF215 *SNPs displayed low *r*^2 ^values of LD (*r*^2 ^= 0.114) suggesting that the association of these SNPs with animal growth are independent and may indicate the presence of *ZNF215 *haplotypes that are associated with animal growth.

The remaining three SNPs showing associations (*P *≤ 0.05) with growth-related traits were the *GRB10 *gene *rs43375833 *SNP (progeny carcass conformation and rump angle), the *PEG3 *gene *rs17871322 *SNP (body depth and animal stature), and the *ZIM2 *gene *rs41899913 *SNP (animal stature). In addition, phenotypic associations with at least one of the carcass or growth-related traits examined approached statistical significance (*P *≤ 0.10) at several SNP loci: three SNPs with progeny carcass fat (*GRB10_p.A5394141C*, *rs42575474 *[*ZNF215 *gene] and *PEG3_p.A64370595G*); one SNP with culled cow carcass weight (*rs42637578 *[*TSPAN32 *gene]); three SNPs with rump width (*GRB10_p.A5394141C*, *PEG3_p.A64370595G*, *rs17871322 *[*PEG3 *gene]); one SNP with animal stature (*GRB10_p.A5394141C*); and two SNPs with body depth (*PEG3_p.C64367437T*, *rs41899913 *[*ZIM2 *gene]). None of the genotyped SNPs were associated with chest width (results not shown).

## Discussion

### Associations between SNPs in candidate bovine imprinted genes and performance traits

The recent availability of whole genome sequences has highlighted the wealth of DNA sequence variation contained within mammalian genomes, the vast majority of which exists as SNPs. The abundance of these genetic polymorphisms coupled with their ease of detection (via DNA sequencing) and ease-of-genotyping has resulted in their adoption as the marker of choice for genotype-phenotype association analyses in livestock genetic studies [5]. Indeed, the recent advent of high-throughput SNP genotyping platforms for livestock, such as the Illumina BovineSNP50 assay [4], has provided animal geneticists with vast quantities of data for association studies performed at a genome-wide level. However, a perceived drawback of such genome-wide association (GWA) studies is the detection of false-positive associations between a SNP and a trait-of-interest which can confound studies, particularly when an associated SNP occurs in a gene or region of the genome displaying no obvious biological connection to the trait [52]. The detection and removal of spurious genotype-phenotype associations in GWA studies requires stringent statistical analysis involving the use of multiple-testing corrections; however, these can significantly reduce the number of associations reported in a study [53]. Furthermore, it is becoming increasingly recognised that correcting for multiple tests using conventional methods can be too restrictive in genotype-phenotype association studies resulting in SNPs displaying true associations being overlooked [54-56].

A commonly used method to circumvent the detection of spurious genotype-phenotype associations is the adoption of candidate gene strategies whereby SNPs are pre-selected for association analyses based on their location within or proximal to genes/loci known to have a molecular role in regulating a phenotype of interest [55,57-59]. Candidate gene approaches are also expected to reduce the number of false-negative genotype-phenotype associations (*i.e*. true associations that are erroneously rejected after rigorous statistical testing) that can also be generated in GWA studies [60,61]. Consequently, in the present study, we have adopted a candidate gene approach by analysing genotype-phenotype associations between SNPs in a panel of eight putatively imprinted bovine genes, one of which (*PEG3*) has been previously shown to be subject to genetic imprinting in cattle. The remaining seven genes have been shown to be imprinted in at least one other mammalian species and therefore may be imprinted in cattle based on the appreciable conservation of imprinting between orthologs from different species [36].

Mammalian imprinted genes have been shown to play a pivotal role in mediating growth and development. This suggests that imprinted genes may serve as candidate loci harbouring potentially important DNA sequence polymorphisms contributing to heritable variation in livestock performance traits--a hypothesis that is supported by a number of recent genotype-phenotype association studies performed in domestic livestock populations [8,21,22,24,26,27,62]. In this study, significant phenotypic associations (*P *≤ 0.05) were detected between SNPs located proximal to or within six of the eight candidate bovine imprinted genes analysed--*CALCR*, *GRB10*, *PEG3*, *RASGRF1*, *ZIM2*, and *ZNF215--*and range of cattle performance traits; significant associations (*P *≤ 0.05) were not observed between performance traits and SNPs within the *PHLDA2 *and *TSPAN32 *genes, although one SNP within the bovine *TSPAN32 *gene showed a tendency to be associated (*P *≤ 0.10) with a number of the performance traits assessed.

It should be stated that in this study we applied a Bonferroni correction [63] in an attempt to minimise the incidence of false-positive associations. However, none of the adjusted genotype-phenotype association *P*-values were significant at the *P *≤ 0.05 level following this correction. Despite this, we believe that the uncorrected *P*-values ≤ 0.05 for the genotype-phenotype associations reported in this candidate gene study are supported by the molecular biological functions of the candidate bovine imprinted genes analysed in this study.

For example, the *CALCR *gene encodes the calcitonin hormone receptor protein--a seven-transmembrane receptor located on the surface of osteoclasts to which calcitonin binds activating adenylate cyclase leading to the inhibition of osteoclastic bone resorption [64]. Previous studies have shown that SNPs in the porcine *CALCR *gene (whose imprinting status has yet to be defined, although preferential maternal expression of this gene has been reported in mouse brain tissue [65]) are associated with osteological development and growth performance in pigs [66,67]. Notably, no significant associations were observed between *CALCR *SNP genotypes and the more direct measures of animal growth in this study (*i.e*. body depth, chest width, rump width, rump angle and animal stature). However, the associations between both bovine *CALCR *SNPs analysed and angularity and body condition (both of which are measures of subcutaneous fat levels in live animals) as detected here does suggest that the *CALCR *locus encompasses or is located proximal to a QTL that contributes to inter-animal differences in bovine body conformation traits, especially those related to fat deposition.

*GRB10 *(or maternally expressed gene 1 [*MEG1*]) encodes an adapter protein which is known to interact with certain tyrosine kinase receptors, such as insulin receptors and insulin-like growth factor receptors [68], and acts to restrict foetal and placental growth during mammalian development [69]. This gene displays preferential maternal expression in the majority of mouse tissues examined to-date, with bi-allelic expression of the human *GRB10 *ortholog in corresponding human tissues and preferential paternal expression in human and mouse brain tissue [16]. Furthermore, perturbations of the imprinting status/gene dosage of *GRB10*, whereby the maternal copy of the *GRB10 *gene has been duplicated, has been shown to result in severe pre- and post-growth retardation in mice [70]. In this study, SNP genotype associations were observed between the bovine ortholog of this gene and angularity, body conditioning score and rump angle--traits related to animal development and growth. Based on these observations in cattle, it is possible that mutations in the *GRB10 *gene sequence alter the ability of the GRB10 protein in restricting foetal growth and development hence leading inter-individual differences in growth.

In mammals, both the *PEG3 *and *ZIM2 *genes form an imprinted gene cluster, a feature common to many imprinted genes [71]. The *PEG3 *gene cluster is located on chromosomes 7 and 19 in mouse and humans, respectively, and consists of at least five differentially-imprinted genes, although analysis of this domain in human, mouse and cow has revealed some species-specific gene rearrangements [33]. The paternally expressed *PEG3 *gene encodes a Krüppel-type zinc finger protein that may play a role in transcriptional regulation [72-74]. Also, the murine ortholog of this gene, *Peg3*, has been shown to be critical in cellular and behavioural functions including cellular proliferation, apoptosis and nurturing behaviour [40,75]. The role of the maternally expressed *ZIM2 *gene is less well understood, but it has been shown to share at least seven upstream exons and a transcriptional start site with *PEG3 *in humans, suggesting some similarities for the function of the *PEG3 *and *ZIM2 *gene products [39]. Two of the seven SNPs within the bovine *PEG3 *gene cluster were associated with animal stature, while one of these two SNPs was also associated with angularity, thus supporting a role in growth for this imprinted domain. In addition, three *PEG3 *domain SNPs were associated with perinatal mortality (with an additional two *PEG3 *domain SNPs displaying a tendency to be associated with this trait), while one *PEG3 *SNP was associated with gestation length, suggesting that the bovine *PEG3 *imprinted genes cluster underlies QTL for calf performance and fertility. Interestingly, aberrant methylation of the *PEG3 *gene (resulting in altered expression) has been observed in cases involving stillbirths and aborted foetuses in humans [76,77] and aborted cloned bovine embryos [78], suggesting that this gene has an important role in embryo and foetal viability and survival.

One bovine *RASGRF1 *SNP was analysed in this study and it displayed associations with milk protein percentage and was the only analysed SNP to be associated with somatic cell count. *RASFGR1 *encodes the Ras protein-specific guanine nucleotide releasing factor 1 protein, which has been shown to play a role in signal transduction and growth and development in mice [79]. Previous analyses performed by us identified this gene as being associated with growth traits in performance-tested Limousin cattle [8]. Although no associations between the single analysed *RASGRF1 *SNP with growth were observed in the current study, the data presented here suggest that this gene may play a role in animal health as indicated by the association with somatic cell score--an often cited indicator of resistance to clinical and subclinical mastitis [80,81]. It is unclear how *RASGRF1 *associates with resistance/susceptibility to mastitis; however, previous work has shown that expression of *RASGRF1 *affects the function of the growth hormone-insulin-like growth factor 1 (GH-IGF-1) axis [79], which can modulate the inflammatory response to mastitis [82].

Finally, we detected associations with a number of growth-related traits and the bovine *ZNF215 *gene, which encodes an alternatively spliced zinc-finger DNA binding protein that is localised in the nucleus. Moreover, the *ZNF215 *protein has been shown to contain both a Krüppel-associated (KRAB) box and SCAN box (*i.e. *SRE-ZBP; CT-fin51; AW-1; Number 18) amino-acid structural domains found Krüppel-like C_2_H_2 _zinc finger DNA binding proteins, both of which act to repress transcription [83-85]. In humans, *ZNF215 *is preferentially expressed from the maternally inherited allele and maps to an imprinted gene cluster on human chromosome (HSA) 11p15.5, the genomic region associated with Beckwith-Wiedemann syndrome (BWS)--a genetic disorder characterised by a range of growth abnormalities, including gigantism [86]. It has been proposed that genetic rearrangements disrupt the normal functioning of the genes located within the imprinting domain on HSA11p15.5 (including *ZNF215*) resulting in the manifestation of the BWS phenotype; however, to-date, no functional *ZNF215 *mutations in BWS patients have been reported [83]. In the current study, two SNPs within the bovine *ZNF215 *ortholog were analysed for associations with performance traits. Both SNPs displayed associations with animal stature and angularity while one *ZNF215 *SNP (*rs42575474*) was associated with milk protein percentage, culled cow and progeny carcass weight, body depth and rump width. These data suggest that DNA sequence variation within the bovine imprinting domain orthologous to HSA11p15.5 located on BTA15 may also harbour important quantitative trait nucleotides (QTNs) that similarly influence animal growth. Indeed, it is possible that mutations in the *ZNF215 *gene may alter the binding affinity of the ZNF215 protein to DNA sequences and hence alter the expression of other genes involved in animal growth and developmental pathways.

With the exception of the *CALCR rs42940189 *SNP (a non-synonymous mutation resulting in the substitution of an asparagine amino acid to an aspartic amino acid, both of which are small polar amino acid residues, at amino acid position 116 of the CALCR protein), the ORF gene model location of the remaining 16 SNPs analysed in this study (*i.e*. two upstream, five intronic, five synonymous coding and four non-coding 3'UTR SNPs) does not immediately suggest that these polymorphisms are functional. However, previous studies have shown that non-coding SNPs can have a regulatory function by altering the efficiency of DNA binding proteins that modulate gene expression. For example, a single G-to-A substitution within a non-coding regulatory region of the 3^rd ^intron of the maternally imprinted porcine *IGF2 *gene has been shown to be the causal mutation for a QTL influencing muscle mass and fat deposition in pigs. It is postulated that the 'A' allele at this locus prevents the binding of a transcriptional repressor protein to the *IGF2 *gene sequence; hence individuals inheriting a sire-derived 'A' allele at this SNP display increased muscle mass and reduced fat content due to over-expression of paternally-derived *IGF2 *mRNA [21,87].

3'UTR sequences of protein-coding mRNA transcripts have been shown to have an important function in regulating post-transcriptional process, such as the transportation of mRNA from the nucleus to cytoplasm, mRNA stability and the efficiency of protein translation [88,89]. This has led some authors to suggest that 3'UTR sequences harbour potentially important DNA sequence variants influencing phenotypes in mammals [90]. This assertion further supported by genetic data from livestock whereby 3'UTR SNPs have been shown to be associated with dairy performance traits in cattle [91,92]. However, while it is tempting to speculate that the non-coding SNPs displaying associations with performance traits in the current study are causal it is more likely that these SNPs are associated (through LD) with causal regulatory mutations (or set of mutations) located proximal to, or within, the genetic loci studied that have not yet been identified.

### Imprinted gene loci as candidates for performance traits in cattle

Recent studies have discussed the evolutionary consequences and of parent-of-origin effects in animal breeding programmes and their effect on quantitative traits, especially where differences exist in the intensity of selection for sex-specific performance traits (*e.g*. muscling and milk traits) and male and female effective population sizes [29]. While previous genome scans for production traits studies using multi-generational structured livestock resource populations/pedigrees have incorporated the effect of imprinting and monoallelic expression [19,22,93-96], the inclusion of parent-of-origin effects in our statistical analysis was not possible as the DNA samples used were derived solely from progeny-tested AI sires. Therefore, it is important to note that the analyses presented here may have reduced sensitivity to phenotypic effects for SNPs associated with imprinted genes.

Furthermore, imprinting is expected to affect the statistical models used for quantitative genetic analyses and animal breeding by causing differences between male and female breeding values and leading to deviations in additive and non-additive genetic effects. For example, in the case of phenotypes influenced by imprinted loci, offspring are expected to phenotypically resemble the parent from which the functional allele has been inherited--an observation that has particular importance in breeding strategies when favourable alleles occur at imprinted loci [28,29]. Notably, in the present study, two of the six genes displaying significant associations (*P *≤ 0.05) with performance traits are inferred to be maternally expressed based on their imprinting status in other species (*i.e*. *CALCR *and *ZNF215*). As the association analyses presented here are based on phenotypic data from progeny-tested AI sires, this leads to the paradoxical observation--contrary to the imprinting model--that variation in maternally expressed genes inherited from a sire are associated with progeny phenotypes. However, this can be resolved by noting the following: (1) that the genetic merit for each of traits examined here is calculated from many descendents across multiple generations (with female intermediaries); therefore, variation in sire-derived paternally imprinted genes could be associated with performance; and (2) that the SNPs associated with performance traits in this study may actually be in LD with causal variants at neighbouring loci.

## Conclusions

The results presented here add to previous investigations performed by us and other groups suggesting that candidate imprinted genes contribute to many performance traits in cattle. These findings, together with the documented biological roles of these candidate imprinted genes suggest that these genes represent an important reservoir of molecular markers for future genetic improvement of dairy and beef cattle populations [18].

## Authors' contributions

DAM, KMS and EWB performed laboratory work including validation of the SNPs analysed in this study, preparation of DNA samples for genotyping, data analysis and drafted the manuscript. DPB collected phenotypic data for the animals used in this study, performed statistical analyses of the genotypic and phenotypic data and contributed to the preparation of the manuscript. DJH and MPM extracted DNA from the semen samples used and also prepared samples for genotyping. RDE contributed to the collection and analysis of the phenotypic data used in this study. CS and DEM conceived the study, participated in its design and coordination and helped to draft the manuscript. All authors read and approved the final manuscript.

## Supplementary Material

Additional file 1 **Descriptions of the performance traits assessed in the present study**. This Microsoft Word file contains detailed information for each of the phenotypic trait analysed as provided by the Irish Cattle Breeding Federation (ICBF) (http://www.icbf.com) Click here for file

Additional file 2 **Within-gene pairwise SNP linkage disequilibrium (LD) values**. This Microsoft Excel file contains *D*' and *r*^2 ^measures of LD for each within-gene pairwise SNP combination Click here for file
